# Supplementary material for: The impact of the COVID-19 epidemic on students’ mental health: A cross-sectional study
Source: PLoS One. 2022 Sep 22;17(9):e0275167. doi: 10.1371/journal.pone.0275167 (PMC9499216; doi:10.1371/journal.pone.0275167)
Supplement: S1 Table — (PDF) [file pone.0275167.s001.pdf]

Associations of scales of depression, anxiety and stress with students socio-demographic variables, their medical studies and health status during the coronavirus pandemic – Results of multivariate logistic regression.

**Variable**	**Multivariate logistic regression**					
	**Serious versus moderate**					
	**Depression scale**		**Anxiety scale**		**Stress scale**	
	OR (95%CI)	P	OR (95%CI)	P	OR (95%CI)	P
**Gender**						
Male	1.00 [a]		1.00 [a]		1.00 [a]	
Female	1.56 (0.91-2.66)	0.107	2.21 (1.09-4.49)	0.027	3.37 (1.70-6.68)	<0.001
**Age**						
≤20	1.00 [a]		1.00 [a]		1.00 [a]	
21-30	1.81 (0.61-5.44)	0.288	0.59 (0.14-2.47)	0.467	1.82 (0.42-7.90)	0.424
31+	0.37 (0.04-3.20)	0.370	0.31 (0.02-5.10)	0.416	0.21 (0.02-2.20)	0.194
**Year of studies**						
First	1.00 [a]		1.00 [a]		1.00 [a]	
Second	0.53 (0.23-1.25)	0.150	0.20 (0.03-1.14)	0.069	0.18 (0.05-0.69)	0.012
Third	0.32 (0.09-1.19)	0.090	0.16 (0.02-1.40)	0.099	0.11 (0.02-0.71)	0.021
Fourth	0.26 (0.06-1.07)	0.061	0.42 (0.04-4.25)	0.461	0.18 (0.02-1.46)	0.109
Fifth	0.12 (0.03-0.49)	0.003	0.16 (0.02-1.49)	0.107	0.09 (0.01-0.71)	0.023
Sixth	0.21 (0.05-0.81)	0.023	0.36 (0.04-3.32)	0.366	0.09 (0.01-0.71)	0.022

**Marital status**						
Single	1.00[a]		1.00[a]		1.00[a]	
Married/Living with a partner	1.05 (0.62-1.77)	0.868	1.34 (0.61-2.98)	0.466	2.01 (0.90-4.48)	0.088
**Religious**						
Yes	1.00[a]		1.00[a]		1.00[a]	
No	1.50 (0.95-2.36)	0.083	1.10 (0.56-2.14)	0.786	1.36 (0.71-2.62)	0.359
**Settlement type**						
Urban	1.00[a]		1.00[a]		1.00[a]	
Rural	1.33 (0.73-2.43)	0.358	1.40 (0.54-3.62)	0.494	0.57 (0.27-1.20)	0.140
**Cohabitation during the studies**						
Alone	1.00[a]		1.00[a]	0.253	1.00[a]	
With family/room mate	1.18 (0.70-2.01)	0.531	1.53 (0.74-3.16)		2.57 (1.31-5.03)	0.006
**State of emergency**						
In Belgrade	1.00[a]		1.00[a]		1.00[a]	
Not in Belgrade/Serbia	1.06 (0.68-1.66)	0.795	0.90 (0.46-1.75)	0.748	0.99 (0.52-1.89)	0.938

**On-line lectures**						
Few	1.00 [a]		1.00 [a]		1.00 [a]	
Moderate	0.96 (0.58-1.61)	0.892	1.64 (0.76-3.56)	0.206	1.03 (0.50-2.13)	0.938
Plenty	0.55 (0.28-1.07)	0.077	0.83 (0.33-2.06)	0.687	0.52 (0.21-1.29)	0.157
**Means of work**						
Yes	1.00 [a]		1.00 [a]		1.00 [a]	
No	0.75 (0.47-1.22)	0.247	0.68 (0.32-1.48)	0.335	0.94 (0.46-1.90)	0.857
**COVID-19 infected**						
Yes	1.00 [a]		1.00 [a]		1.00 [a]	
No/not sure	0.88 (0.54-1.44)	0.617	0.99 (0.48-2.02)	0.974	0.80 (0.40-1.60)	0.530
**General health**						
Poor	1.00 [a]		1.00 [a]		1.00 [a]	
Average	/	0.998	/	0.998	/	0.999
Good	/	0.998	/	0.998	/	0.999
**Physical health**						
Poor	1.00 [a]		1.00 [a]		1.00 [a]	
Average	1.23 (0.26-5.92)	0.795	3.79 (0.52-27.49)	0.187	/	0.998
Good	0.71 (0.14-3.57)	0.683	2.80 (0.36-21.68)	0.324	/	0.998
**Mental health**						

Poor	1.00 [a]		1.00 [a]		1.00 [a]	
Average	0.06 (0.01-0.50)	0.009	/	0.996	/	0.996
Good	0.01 (0.01-0.07)	<0.001	/	0.996	/	0.996

a Reference category

Associations of scales of depression, anxiety and stress with students socio-demographic variables, their medical studies and health status during the coronavirus pandemic – Results of multivariate logistic regression with significant predictors in bivariate logistic regression.

**Variable**	**Multivariate logistic regression**					
	**Serious versus moderate**					
	**Depression scale**		**Anxiety scale**		**Stress scale**	
	OR (95%CI)	P	OR (95%CI)	P	OR (95%CI)	P
**Gender**						
Male			1.00 [a]		1.00 [a]	
Female			1.98 (1.04-3.77)	0.038	2.62 (1.44-4.77)	0.002
**Age**						
≤20	1.00 [a]		1.00 [a]		1.00 [a]	
21-30	1.58 (0.53-4.66)	0.408	0.30 (0.08-1.16)	0.082	0.99 (0.25-3.80)	0.983
31+	0.45 (0.62-3.31)	0.434	0.21 (0.02-2.66)	0.229	0.22 (0.03-1.63)	0.138
**Year of studies**						
First	1.00 [a]		1.00 [a]		1.00 [a]	

Second	0.60 (0.26-1.38)	0.234	0.22 (0.04-1.13)	0.070	0.29 (0.09-0.92)	0.036
Third	0.44 (0.12-1.55)	0.201	0.38 (0.05-2.81)	0.346	0.33 (0.60-1.77)	0.194
Fourth	0.42 (0.11-1.57)	0.197	0.94 (0.11-7.76)	0.953	0.60 (0.10-3.70)	0.581
Fifth	0.18 (0.05-0.69)	0.012	0.31 (0.04-2.29)	0.251	0.33 (0.06-1.88)	0.210
Sixth	0.33 (0.09-1.18)	0.090	0.61 (0.08-4.56)	0.634	0.28 (0.05-1.51)	0.139
**Marital status**						
Single						
Married/Living with a partner						
**Religious**						
Yes	1.00 [a]					
No	1.43 (0.92-2.22)	0.109				
**Settlement type**						
Urban						
Rural						
**Cohabitation during the studies**						
Alone					1.00 [a]	
With family/room mate					2.27 (1.26-4.06)	0.006

**State of emergency**						
In Belgrade						
Not in Belgrade/Serbia						
**On-line lectures**						
Few						
Moderate						
Plenty						
**Means of work**						
Yes	1.00 [a]					
No	0.71 (0.45-1.12)	0.144				
**COVID-19 infected**						
Yes						
No/not sure						
**General health**						
Poor						
Average						
Good						
**Physical health**						

Poor	1.00 [a]					
Average	0.62 (0.15-2.44)	0.488				
Good	0.34 (0.91-1.30)	0.116				
**Mental health**						
Poor	1.00 [a]					
Average	0.07 (0.01-0.52)	0.010				
Good	0.10 (0.01-0.07)	<0.001				

a Reference category
